# Optimization of parameters affecting corn cob activated carbon in the removal of cationic brilliant green dye from aqueous solution

**DOI:** 10.1038/s41598-026-45204-x

**Published:** 2026-04-17

**Authors:** Mizbawu Kanea Gurmu, Mohamed Asanu Mokonon

**Affiliations:** 1 Department of Environmental Engineering, College of Engineering and Technology, Bule Hora University, P.O. Box: 144, Bule Hora, Ethiopia; 2 Department of Chemical Engineering, College of Engineering and Technology, Bule Hora University, P.O. Box: 144, Bule Hora, Ethiopia

**Keywords:** Adsorption isotherms, Activated carbon, Corncob, Optimization, Removal efficiency, Chemistry, Environmental sciences

## Abstract

Excessive release of dye pollutants from textile and pulp industries becomes a major water pollution source. These toxic pollutants are extremely toxic to living things. The present paper deals with the preparation of a novel low-cost and eco-friendly chemically treated corncob activated carbon as an adsorbent and were further explored for the removal of brilliant green (BG) dye from an aqueous solution. The adsorption process is the most effective technique for the removal of dye from wastewater. The activated carbon was produced using H_2_SO_4_ activation. Batch experiments were performed on synthetic wastewater, and the effect of operating parameters such as pH, initial concentration of dye, contact time, and adsorbent dosage were studied. The maximum removal efficiency of brilliant green dye using corncob activated carbon was optimum at pH (7.172), adsorbent dosage (0.276 g), contact time (63.847 min), and initial concentration (59.463 mg/L). The potential of corn cob-activated carbon and agricultural waste for dye removal from wastewater clearly aligns with future goals and requires collaborative efforts from both researchers and industry professionals to develop a visible, economical, and viable water treatment technology.

## Introduction

Pollution due to rapid population growth and industrial development has largely contributed to water quality contamination^1^. Ecosystems are harmed by the discharge of domestic, industrial, and agricultural wastewater that has not received proper treatment. The issue is even more problematic when it comes to industrial effluents, which have a much more pronounced hazardous nature^2^. Dyes are colored organic compounds used to color various substances like fabrics, paper, food, hair, drugs, etc. Serious environmental issues are caused by the discharge of dyes into wastewater from the textile, cosmetic, paper, and coloring industries. Even a modest amount of dye in the water is undesirable and noticeable. Water bodies with colored surfaces have improper solar penetration, slower photosynthesis, stunted growth of aquatic life, and altered gas solubility. Brilliant green (BG) is a cationic dye that is widely used in the pulp and paper and dyeing industries. When compared to anionic dyes, cationic dyes are more hazardous. Due to its high solubility (100 g/L), BG, a commonly used dye, moves to incorporate the aquatic systems; additionally, because of its high volatility, it pollutes the atmosphere^3^. The triphenylmethane family’s "bright green BG," an organic dye, appears as a golden crystal^4^. Brilliant green (BG), often called emerald green or malachite green G, is a dye made of daimio-triphenylmethane^4^. Hence, BG has been linked to causing several health-related disorders, for example, skin and respiratory tract irritation, coughing, shortness of breath, and irritation to the gastrointestinal tract, with symptoms of nausea, abdominal pain, and diarrhea. Therefore, the removal of BGD before their discharge into the environment is crucial and mandatory. Different methods are available for the removal of dyes from water. These are physical, chemical, and biological methods. A lot of work has been put into finding ways to degrade color impurities in industrial effluents since they pose a serious threat to both human health and the environment. One such option is employing agricultural waste as an adsorbent^5^. Adsorption is a very effective physical separation technique in terms of initial cost, simplicity of design, ease of operation, and insensitivity to toxic substances^6^.

**Fig. 1:**
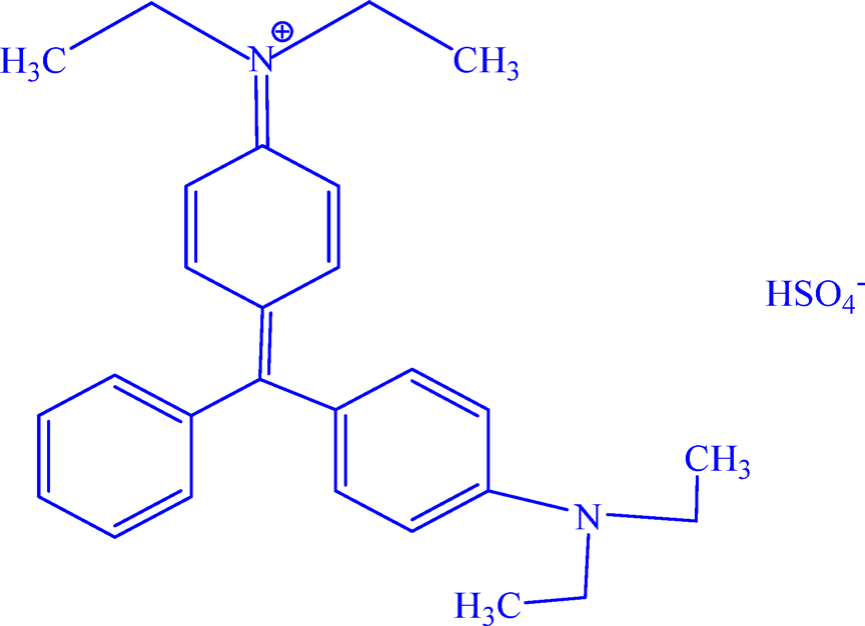
Molecular structure of BGD. *Source*^14^:.

Adsorption offers a desirable option for treatment, particularly if the adsorbent is inexpensive and easily accessible. The remarkable adsorption effectiveness of activated carbon (powdered or granular) for organic molecules, even from dilute solutions, gives the adsorption process an edge over the other methods, although commercially available activated carbons are very expensive. Commercial activated carbon is an efficient adsorbent for removing dyes due to enough surface area and adsorption capacity. Many researchers are increasingly more interested in finding alternative, inexpensive adsorbents from agricultural waste to remove organic contaminants.AC has been produced using a wide range of high carbon content materials, including wood^7^, coal^8^, peat^9^, nutshells^10^, sawdust^11^, bones, husk, and others, with varying degrees of success^12^. However, they did not address the use of corn cob-activated carbon as an adsorbent for the removal of BGD from an aqueous solution. Corncobs, also known as Zea mays cobs, are biomass by-products of corn production. The purpose of this work is to activate corncob and transform it into a viable adsorbent for removing contaminants from an aqueous solution. The treated corncob performed better than the untreated corncob, offering a promising alternative for the removal of dye from diluted industrial effluents^13^. The activation is carried out using H_2_SO_4_ activation. The main purpose of this work was to optimize the adsorption capacity of locally available low-cost adsorbent corncobs for the removal of cationic BGD from an aqueous solution. Improved removal efficiency and optimization of the parameters were studied (Fig. 1).

## Materials and methods

### Raw materials and chemicals

In this study, the raw materials were collected from local farmers of Bule Hora. The laboratory experiments were conducted at the Chemical Engineering, Biology, and Chemistry Department at Bule Hora University, and all chemicals used are analytical grade. The chemicals, reagents, and materials required for the experimentation were sulphuric acid and sodium bicarbonate and brilliant green powder (C_27_H_34_N_2_O_4_S, AR 85 Merck). Distilled water, sodium chloride, hydrochloric acid, and sodium hydroxide.

### Instruments

The apparatus used was a ceramic crucible, conical flasks, measuring cylinders, a funnel, the ceramic crucible, beakers, Whitman filter paper, a hot plate, a blast drying oven (DHG-9055A), a box-type resistance furnace (SX-2.5–12), a refrigerator, a fume hood (SW-TFG-12), a knife, a mortar and pestle (grinder), a shaker and beaker, an electronic balance (AS220-R2), a pH meter (pH-016), X-ray diffraction (XRD-700), FTIR, and SEM are used to characterize the prepared CCAC, and a UV–Visible double beam spectrophotometer (UV-5000) is used to read the absorbance (Fig. 2).

#### Sample preparation and experimental procedures

**Fig. 2:**
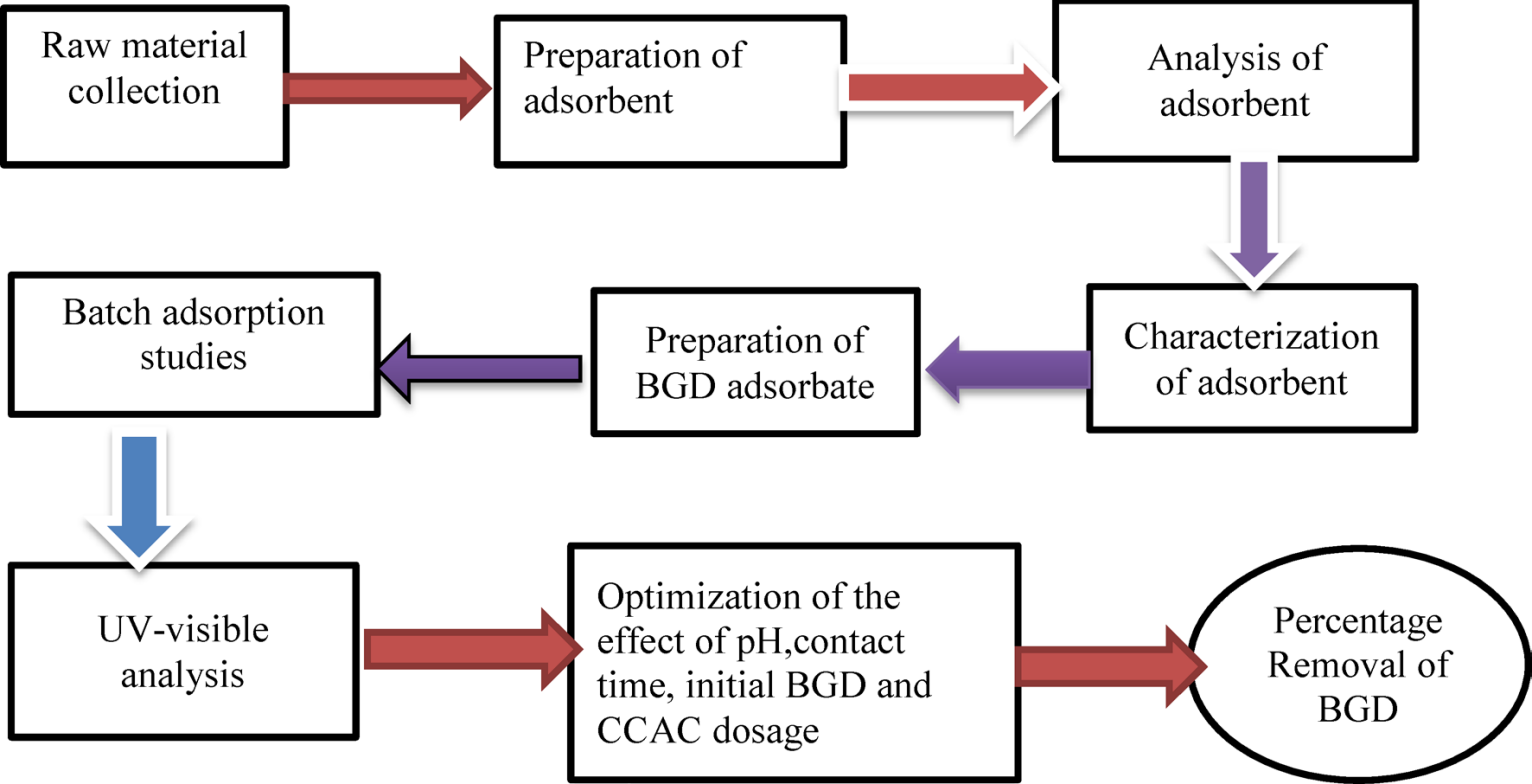
Flow scheme of methodology.

### Sample preparation methods

The optimum temperature for corn-activated carbon was 450 °C^15^. After cooling, the reaction mixture was filtered out using distilled water and soaked in 1% NaHCO_3_ to neutralize any remaining acid. The beaker was covered for 24 h to let the charcoal.

Then it was washed with distilled water until a neutral pH of between 6 and 7 was obtained, or near neutral. Finally, the sample was dried in an oven overnight for 12 h at a temperature of 105 °C. Later, it was sieved to 1–2 mm and kept in a glass bottle until used.

### Preparation of corncobs activated carbon

The collected corncobs were washed using distilled water. The washed corncobs were dried in the sun for 2–3 days and/or dried in an oven at 105 °C to remove the moisture content. Then, the dried corncobs were cut into different sections, such as longitudinal sections. Activation was done with 1 M H_2_SO_4_. Then, 100 g of ground corncobs were weighed into a clean, dry beaker containing 200 ml of H_2_SO_4_ for about 24 h, which was followed by refluxing in a fume hood for 4 h. The paste was obtained and filtered, and the solid phase was dried in an oven at 105 °C for 24 h^16^. To make charcoal, the dried small pieces of corn cobs were put into a furnace to heat at a temperature of 450 °C.

The optimum temperature for corn-activated carbon was 450^15^. After cooling, the reaction mixture was filtered out using distilled water and soaked in 1% NaHCO_3_ to neutralize any remaining acid. The beaker was covered for 24 h to let the charcoal.

**Fig. 3:**
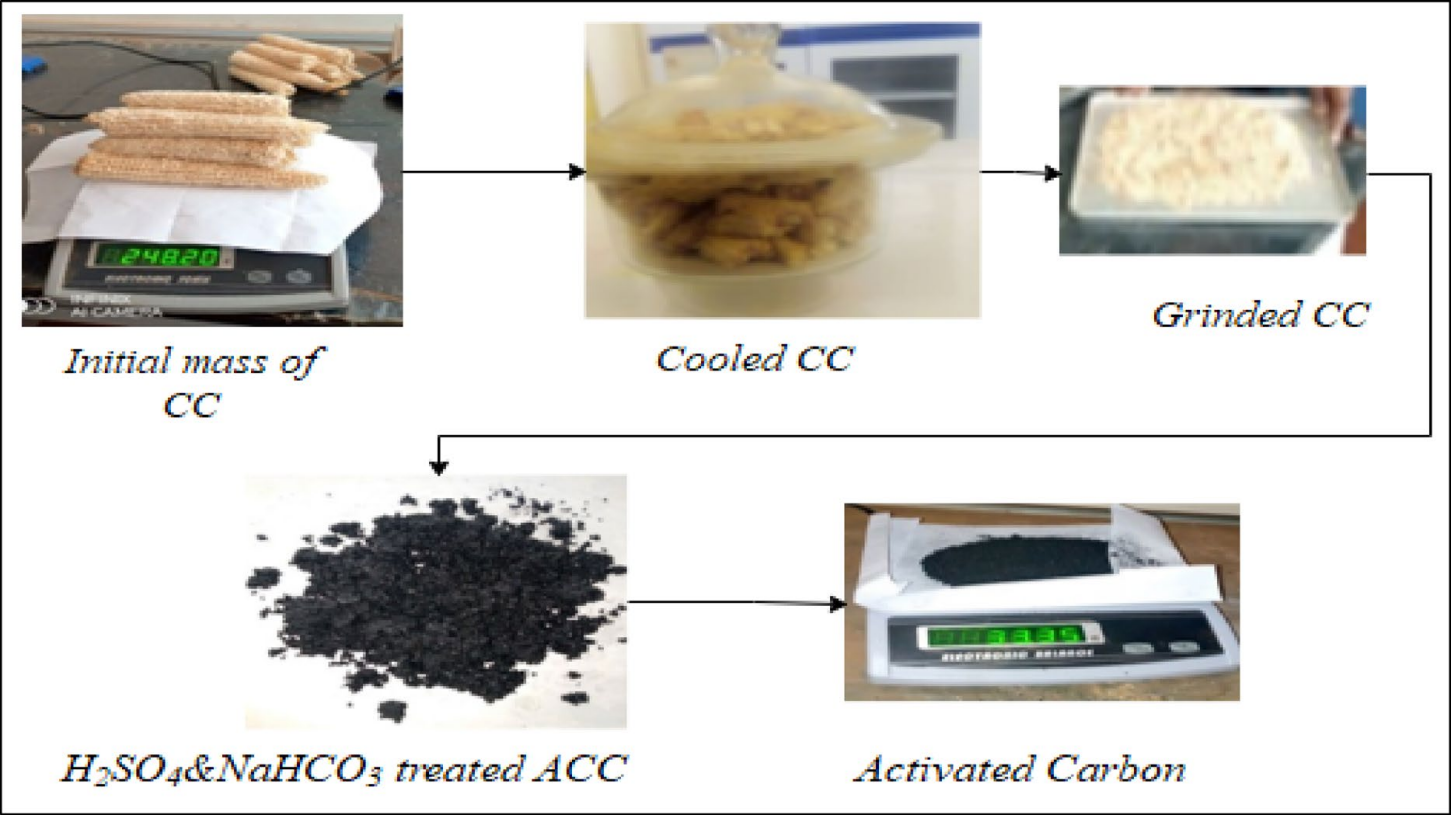
Diagram showing preparation of CCAC^15^.

Then it was washed with distilled water until a neutral pH of between 6 and 7 was obtained, or near neutral. Finally, the sample was dried in an oven overnight for 12 h at a temperature of 105 °C. Later, it was sieved to 1–2 mm and kept in a glass bottle until used (Fig. 3).

### Preparation of BGD adsorbate

The stock solution of 1000 ppm was prepared by dissolving an accurately weighed amount of BG dye in 1 L of distilled water. The stock solution was further diluted using a dilution equation for the experimental solution of desired concentration using distilled water^16^. In this study, 1 g of BGD was dissolved in 1 L of distilled water to prepare 1 g/L of stock solution.

A magnetic stirrer ensured homogenization of the solution. The concentrations of BGD used were from 10 to 150 mg/L^17^. In this study, to determine the standard calibration equation, five series of standard BGD solutions (10, 30, 50, 70, and 90 mg/L) were prepared by diluting the intermediate solution of BGD with distilled water. A blank solution and working standards were run in a UV–Vis spectrophotometer at a maximum wavelength of 625 nm^14^, and five-point calibration curves were established. Then, sample solutions were taken to the UV–Visible spectrophotometer, and direct readings of the sample absorbance were recorded.

Finally, the linear relationship of absorbance versus concentration was plotted, and equations for final concentrations were found from the slope of the graph^18,19^.

1 $$\mathrm{Absorbance}=\mathrm{Slope}*\mathrm{Ce}+\mathrm{Y}-\mathrm{intercept}$$

### Batch adsorption studies

Batch Adsorption Studies: Adsorption studies were carried out by determining the solution pH, initial dye concentration, adsorbent dosage, and contact time. The solution pH was adjusted by adding either 0.1 M NaOH or 0.1 M HCl solutions before the adsorption experiment and carried out at 25 °C.

The adsorption capacity of the BGD dye was calculated using the following expression.

2 $${q}_{e}=\frac{{C}_{o}-{C}_{e}}{w} *V$$

3 $$\mathrm{RE}=\frac{{C}_{o}-{C}_{e} }{{C}_{o}}*$$

where RE is removal efficiency, C0 (mg/L) is the initial concentration of BGD, Ce (mg/L) is the equilibrium liquid phase concentration of BG, V is the volume of the solution (L), and w is the amount of CCAC used in grams^20^.

### Experimental design and statistical analysis

In this study, data analysis was employed using the Design-Expert version 13.1 software tool by RSM using Central Composite Design (CCD) to enhance the BGD decolonization by optimizing the initial operating parameters.

Four main parameters were chosen in order to analyse the process parameters: the initial BGD dye concentration (10–90 mg/l), the pH (2–14), the contact time (20–100 min), and the CCAC adsorbent dosage (0.1–0.5 g/L). Varieties of parameters were selected by taking into account previous relevant efforts^21,22^. The removal efficiency of BGD was the answer (Table 1).

**Table 1:** Specified parameters’ experimental levels using RSM with the central composite design.

Parameter	Units	Low	High	− alpha	+ alpha
CCAC dosage	g/L	0.2	0.4	0.1	0.5
Contact time	Min	40	80	20	100
Initial concentration of BGD	mg/L	30	70	10	90
pH		5	11	2	14

To evaluate and fit the second-order polynomial equation, the following formula was utilized. Through research in laboratories, the ideal circumstances determined through the CCD model were confirmed. According to statistics, the following model equation yields the ideal conditions^23^.

4 $${\mathrm{y}} = \beta {\text{o + }}\sum\limits_{{{\text{j = 1}}}}^{{\mathrm{k}}} {\beta {\mathrm{jXi}}} {\text{ + }}\sum\limits_{{{\text{j = 1}}}}^{{\mathrm{k}}} {\beta {\mathrm{jXi}}^{{\mathrm{2}}} } {\text{ + }}\sum\limits_{{{\text{j = 1}}}}^{{\mathrm{k}}} {\sum\limits_{{{\text{i = 1}}}}^{{\mathrm{k}}} {\beta {\mathrm{jjXjXi}}} }$$

where Xi stands for each parameter, k is the number of variables, and β0, βi, βij, and βii are the coefficients of a polynomial equation of zero order, first order, interaction term, and second order, respectively. Analysis of variance (ANOVA), correlation coefficient (R^2^), F-test values, and a comparison between the experimental and model were used to analyse the results^24^.

## Results and discussions

### Characterization of CCAC

#### Analysis of the point of zero charge

**Fig. 4:**
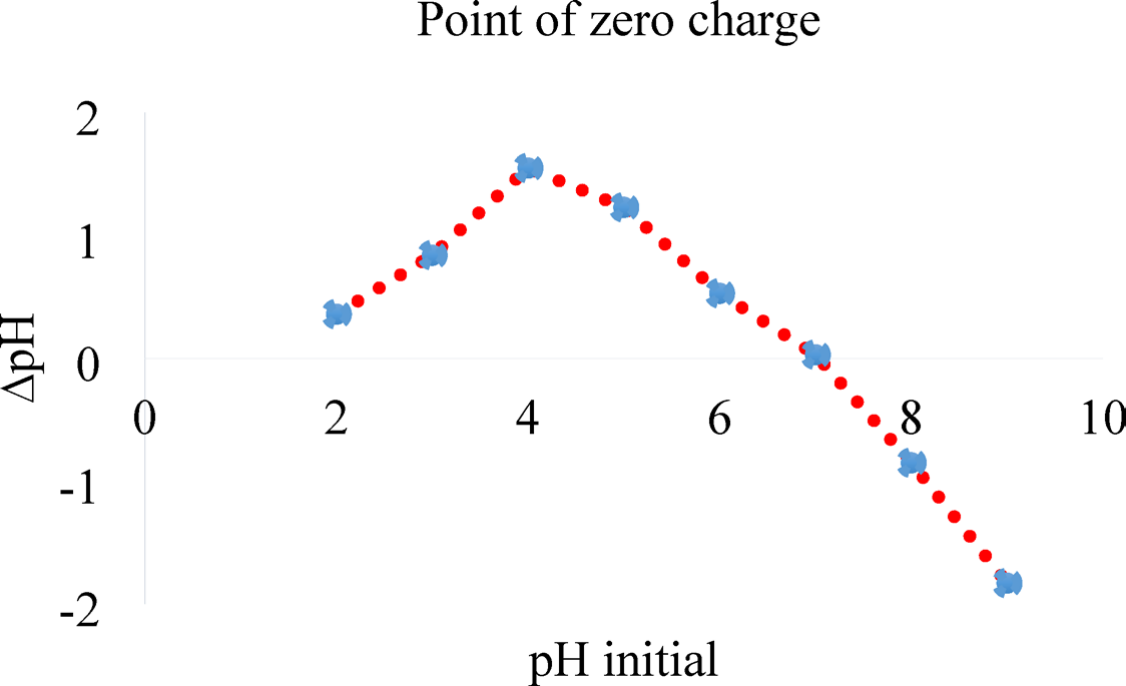
Point of zero charge analysis.

CCAC’s pH of the point of zero charges (pHpzc) was determined using the drift approach depicted in Fig. 4. It was proposed that adsorption of cationic dye on adsorbent is appropriate if pH > pHpzc, while adsorption of anionic species is more advantageous if pH.

The pH drift was proposed as a suitable method for charcoal and is a fast and accurate way to find the point of zero charges^25^. In order to comprehend the change in the surface character of the synthesized adsorbent under varying pH values, the pHpzc of the CCAC was found to be approximately 6.93 in this investigation.

#### X-ray diffraction (XRD)

**Fig. 5:**
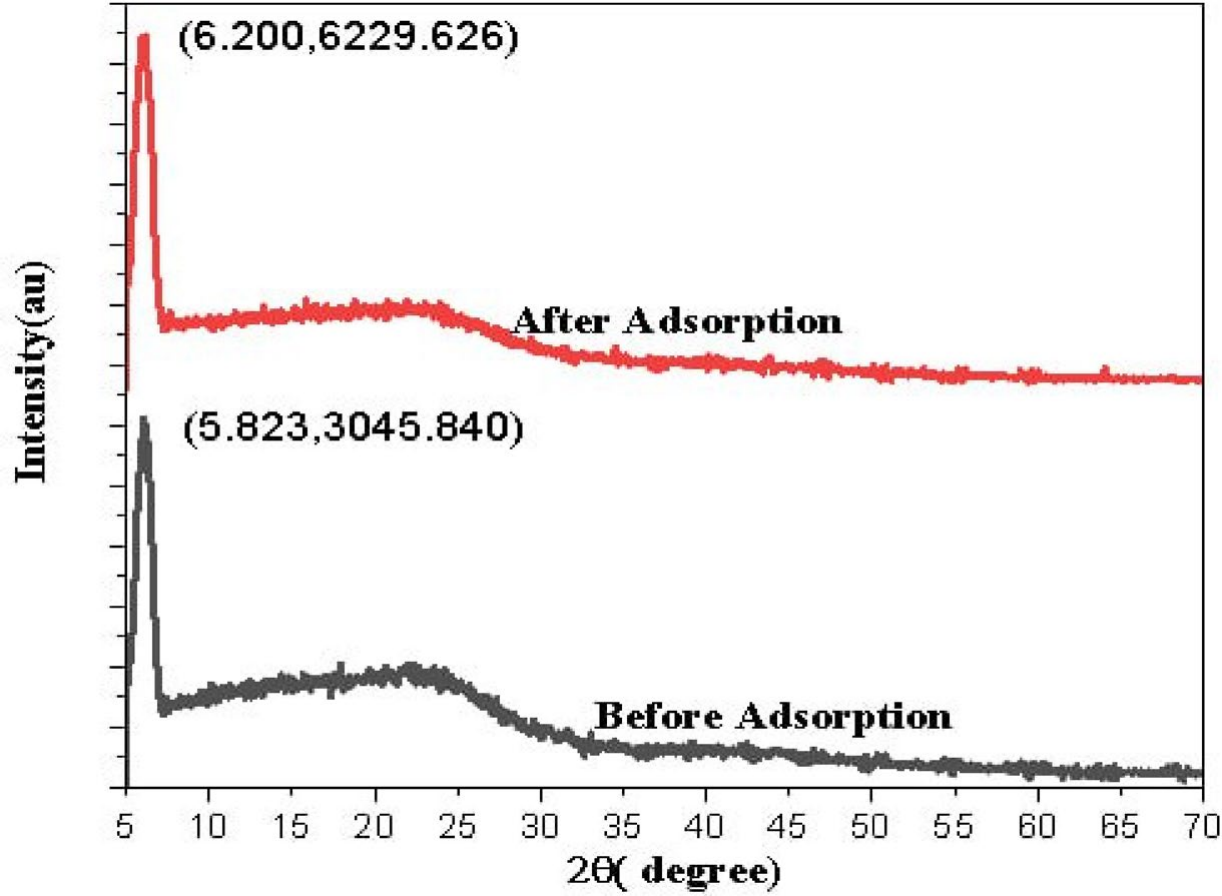
XRD plots of CCAC before and after adsorption.

Figure 5 shows that the powdered CCAC’s structure was primarily amorphous^26^. Maximum reflection occurs at (5.834, 3045.840) before adsorption and (6.200, 6229.629) after adsorption. Following the adsorption of BGD, the diffraction of CCAC marginally reduces, according to the XRD plots^26^. The high lignin content (an amorphous substance) in corncob-activated carbon was the cause of its amorphous content. Activated carbon’s more amorphous portion makes it a better absorbent for removing organic dyes from water and wastewater^27^.

#### Scanning electron microscopy (SEM)

**Fig. 6:**
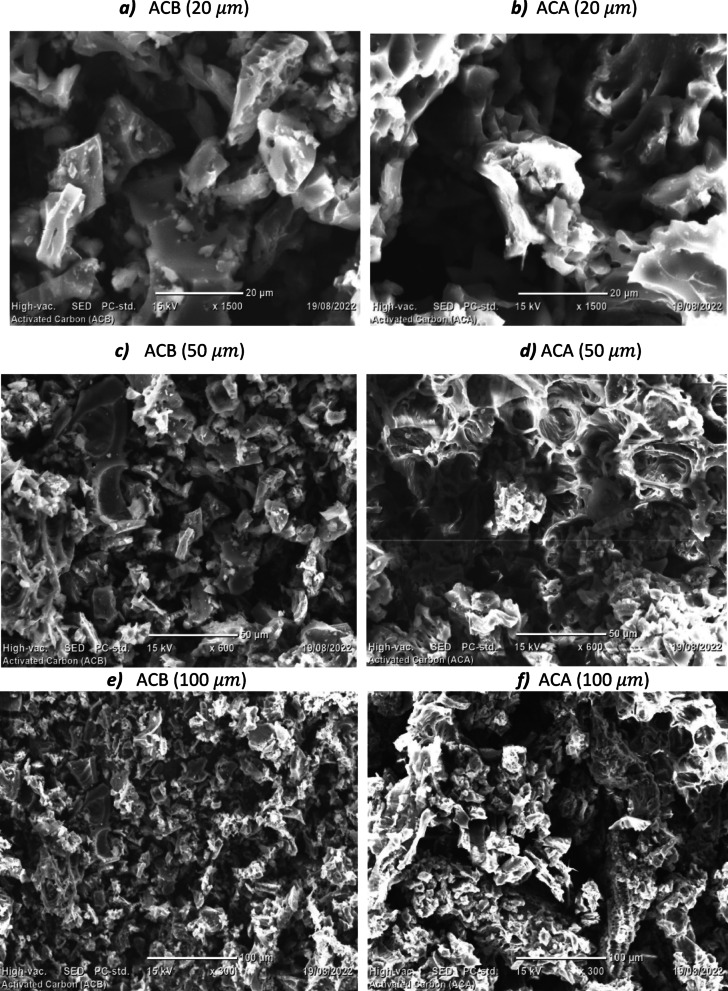
SEM analysis of ACB and ACA samples before and after adsorption.

Figure 6 shows the SEM images of the activated carbon samples both prior to and following adsorption (ACB and ACA). It was shown that the activated carbon had more holes and porous surfaces prior to adsorption than it did following adsorption, which permits adsorption. The smoother surface shape shown in the SEM images of CCAC after adsorption may be due to the concentration of BGD on the adsorbent’s surface^18^.

According to SEM data, there are rough pores of various sizes and shapes on the outside of both ACB and ACA. The wide surfaces on ACA indicate that the adsorbent is saturated, while the small, elongated pores on the ACB indicate that it is suitable for adsorption^18^. Additionally, it is clear from Fig. 6 that the surface of CCAC exhibits substantial surface morphology and becomes smoother and denser following the adsorption of BGD.

#### Fourier transform infrared (FT-IR) spectrum

**Fig. 7:**
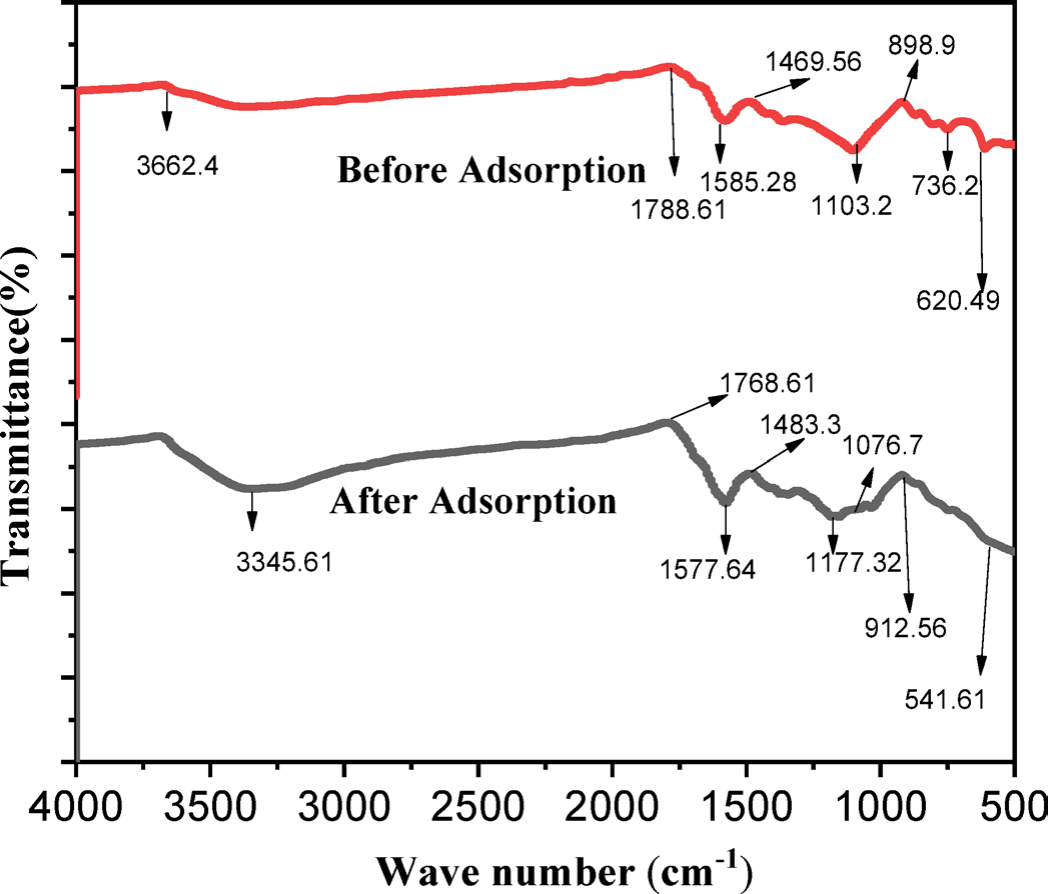
FT-IR spectra of synthesized CCAC before and after BGD removal.

FTIR spectra have been recorded to detect the interaction between adsorbent structures (Fig. 7). The spectra illustrate various peaks at which CAC appear at different wavenumbers recorded in the range of 4000–500 cm⁻^1^ (Table 2).

**Table 2:** Frequencies and functional groups on the surface of CAC (source: FTIR characterization data)^15^.

Frequencies (cm^−1^)	Bond
3000–2800	C–H vibrations in Aliphatic
1770–1650	vibration in C=O
1700–1600	C=C olefin structures
1480–1420	C–H aliphatic structures
1430–1360	bend O–H and C–H hydroxyl
1120–1070	stretch C–O secondary hydroxyl
1060–1100	stretch C–O primary hydroxyl
3664.32	Alcohol
1585.28 and 1469.56	primary and secondary amines (N–H) deformations
1076.2(primary),1177.32,1130.2	C–N stretching of amines

These justify that the surface of CCAC is ionic and preferable for adsorption of BGD. H_2_SO_4_ activation also leads to the incorporation of the sulphur element into the structure of carbon. Hence, the presence of hydroxyl functional groups is the determinate in facilitating the adsorbent. Generally, the wavelength of the adsorbent decreased after adsorption, which means that the surface adsorbent is occupied by the dye.

### Adsorption parameters’ affect

#### Effect of pH for BGD elimination

**Fig. 8:**
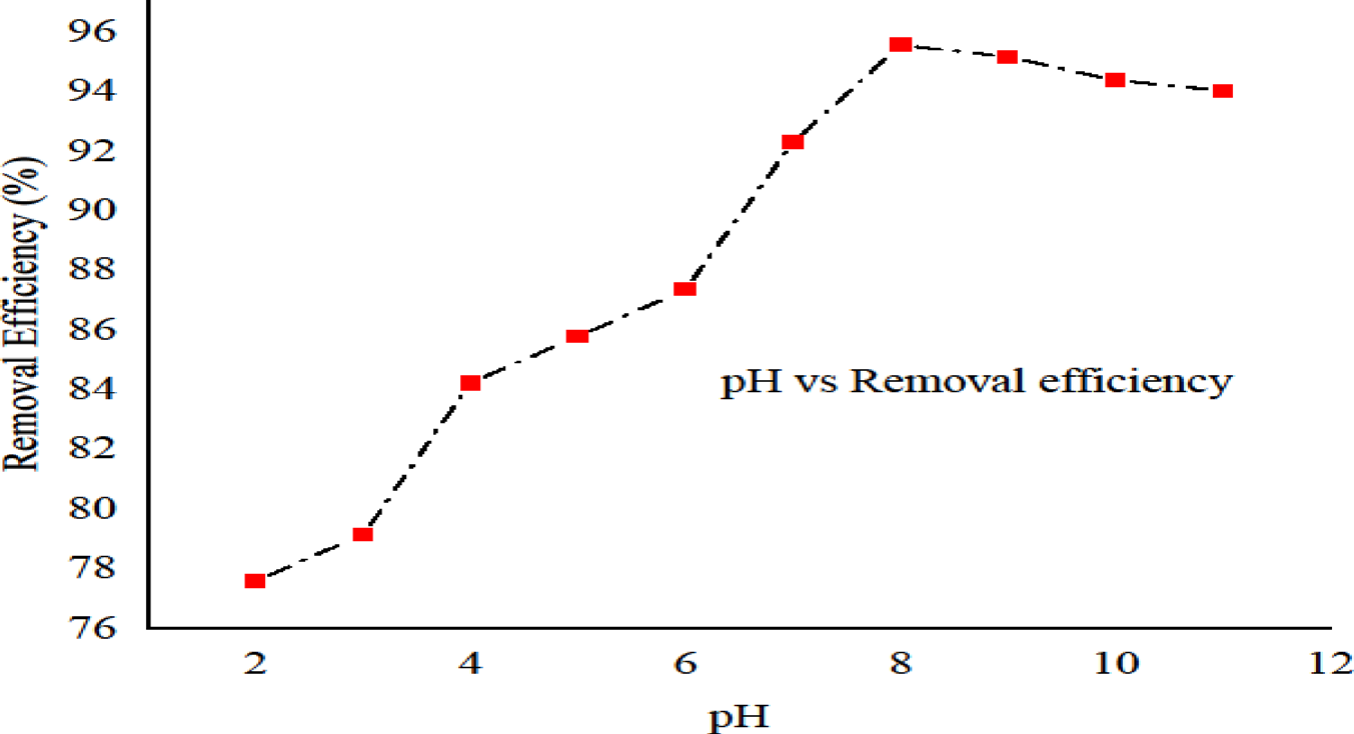
Effect of pH on BGD removal efficiency.

At room temperature, the effect of pH solution on BGD removal was evaluated using 100 mL of 40 mg/L beginning BGD solution in the pH range of 2–12, 0.4 g of adsorbent, 200 rpm shaking speed, and 40 min of contact time. Figure 8 shows that the pH solution has the biggest impact on the removal of BGD from CCAC; at pH 7.8, the maximum adsorption capacity of 95.7% was reached, after which it started to decline.

The fact that CCAC’s adsorption capacity increases as pH rises indicates that the adsorbent’s surface is becoming more negatively charged, which deprotonates the functional groups that are present in CCAC. Deprotonated functional groups serve as locations where cationic BGD can bind. The rivalry of protons with dye molecules present at the adsorbent’s active sites may be the cause of the decreased adsorption capacity at extremely low pH^28^. Rehman et al.^14^ found that raising the pH from 1 to 10 increased the dye removal percentage from 55.94 to 90.93%. At a pH of 8, the maximum adsorption capacity is 9.57 mg/g. Therefore, the pH level of the solution has a significant impact on the process of adsorption of BGD and needs to be taken into account.

#### The impact of contact time

The most important factor influencing the adsorption process is contact time. Batch studies were conducted at room temperature over 20–100 min at 10-min intervals to ascertain the time required to reach equilibrium. The studies used 100 ml of 40 mg/L initial BGD solutions with an adsorbent dosage of 0.3 g, a pH of 8, and a shaking speed of 200 rpm.

Figure 8 above shows the experimental result for the effect of contact time.

**Fig. 9:**
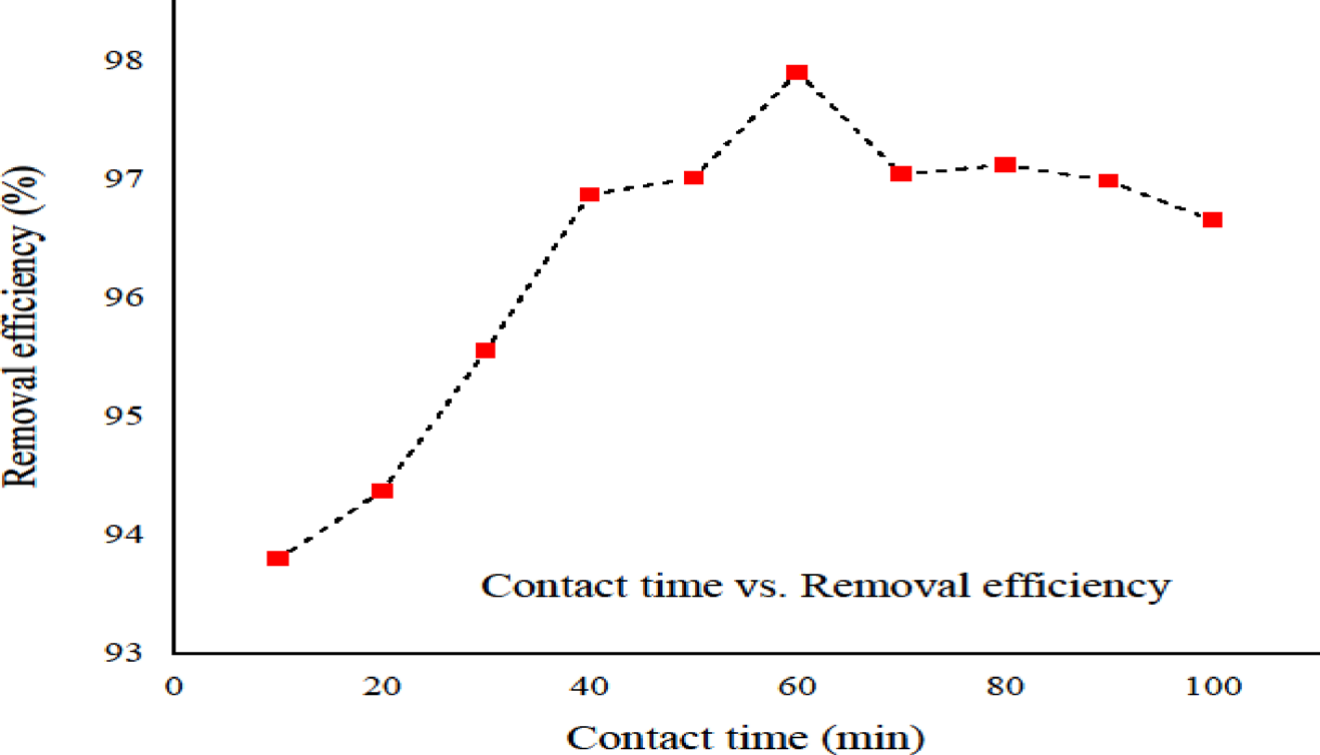
Effect of contact time on BGD removal efficiency.

As seen in Fig. 9 above, the adsorption capability of CCAC decreases significantly as the contact period increases (20–60 min).

It was found that around 94.37% of BGD was eliminated in the first 20 min of adsorption, suggesting that BGD adsorption onto CCAC proceeded quite quickly in the early stages of the adsorption process. BGD was removed quickly due to the adsorbent’s surface having a significant number of free negatively charged active sites. This result is consistent with data from Abbas’s^29^ studies. This suggests that there are significantly more active sites on the adsorbent surface during the initial minutes of adsorption than there are sites elsewhere.

#### Effect of CCAC dosage

By adjusting the adsorbent dose (0.1, 0.2, 0.3, 0.4, and 0.5 g) with an initial BGD concentration of 40 mg/L, the impact of the adsorbent dose on the removal of cationic BGD from an aqueous solution was investigated at room temperature. The pH of the solutions was 8, and they were shaken for 40 min at 200 rpm. Following each sample’s filtering with Whatman filter paper, the BGD ion residuals were finally investigated.

**Fig. 10:**
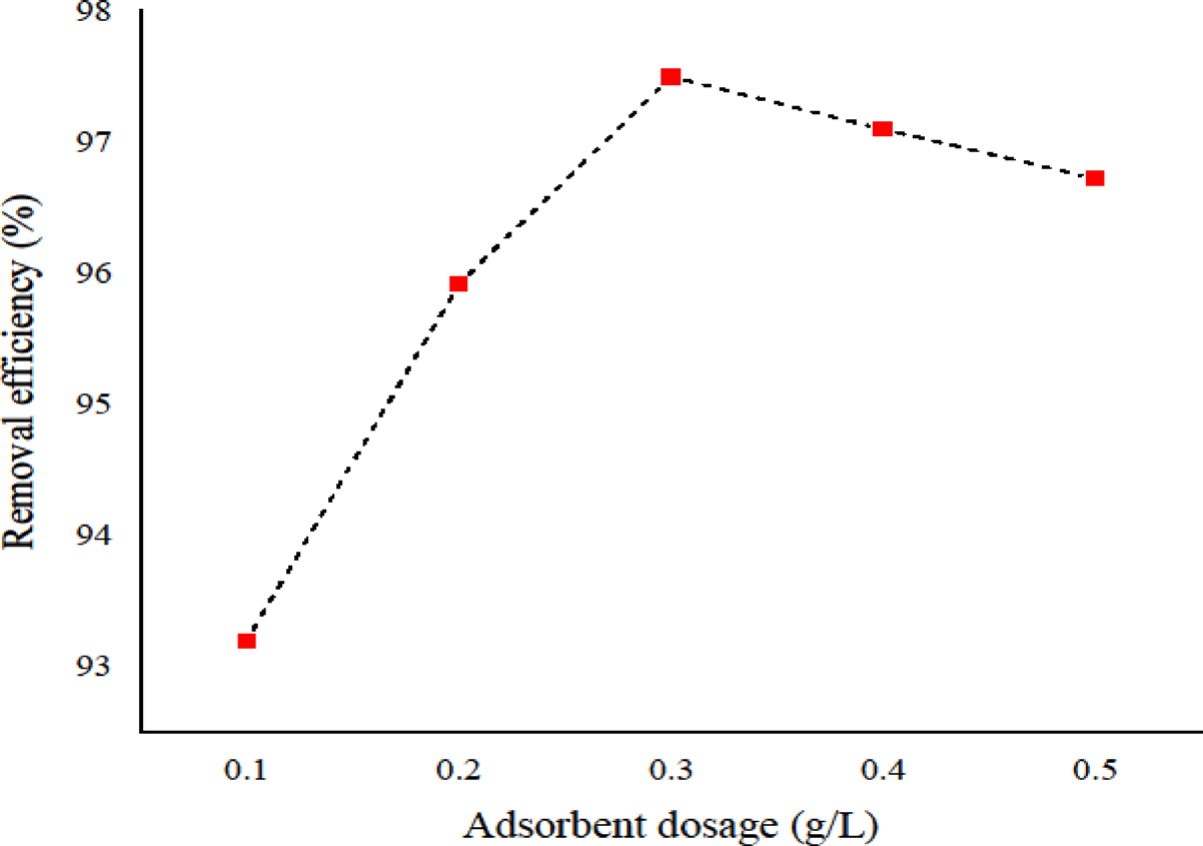
Effect of adsorbent dosage on BGD removal.

The results of the study indicate that the adsorbent dose had a particularly important effect on the removal of BGD, as illustrated in Fig. 10. As the amount of CCAC was increased from 0.1 to 0.3 g, the percentage removal of BGD increased from 93.2 to 98.05%, according to the investigation.

As the dosage was raised, the adsorption capacity dropped. At 0.1 g, the highest adsorption capacity (qe) was 37.35 mg/g. This was in accordance with the research of Tcheka et al.^30^.

#### Initial concentrations effect

While keeping other parameters constant, such as the adsorbent dose of 0.3 g, contact time of 60 min, and pH of 8, the effect of initial BGD concentration on the removal of BGD ions from aqueous solution by CCAC was examined at room temperature by varying the initial concentration (10–90) at intervals of 10 mg/L.

**Fig. 11:**
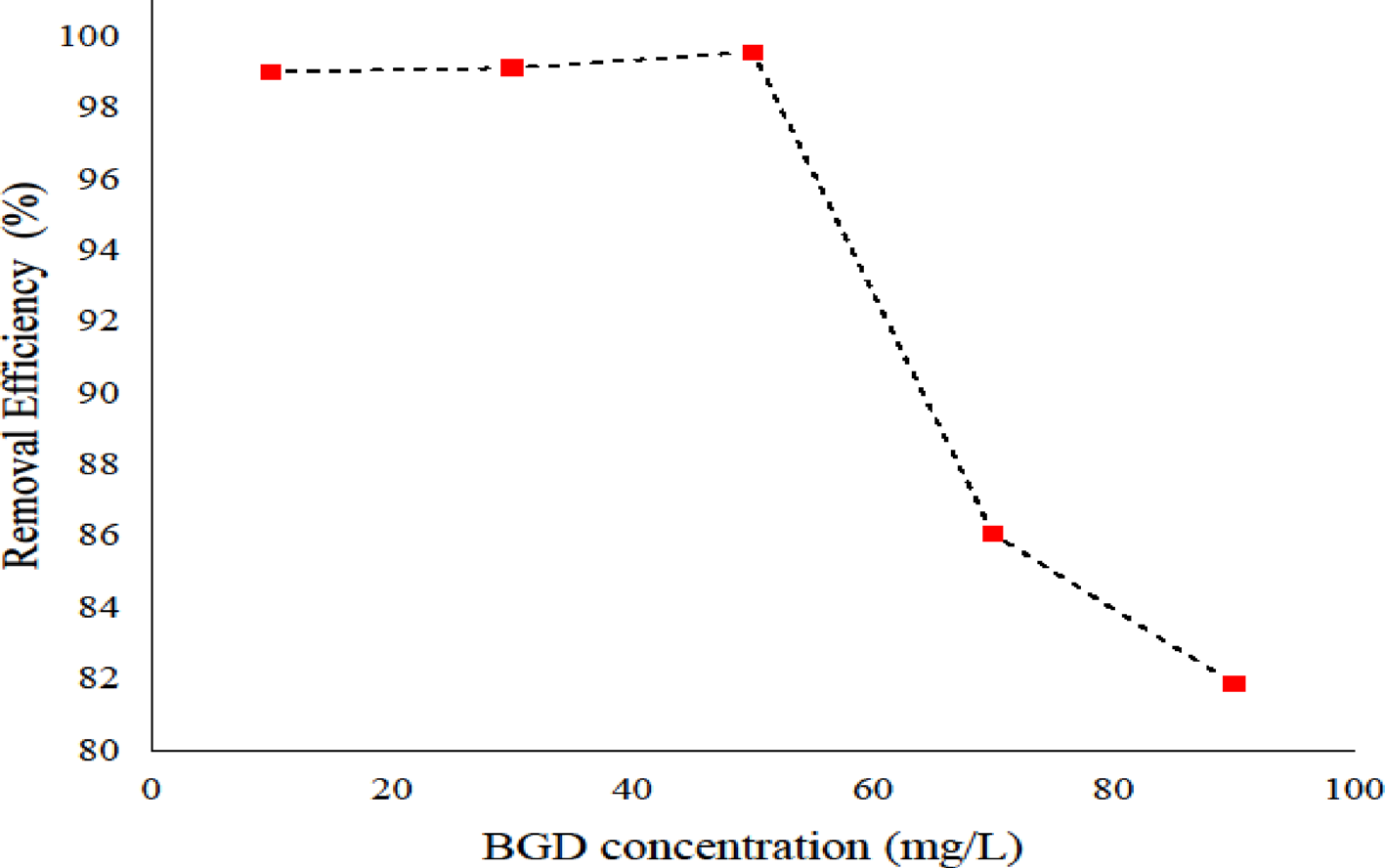
The effect of initial concentrations.

The effects of initial BGD concentration on BGD removal are shown in Fig. 11. It was observed that a lower percentage of BGD was eliminated when the initial concentration of BGD was increased. At the lowest BGD concentration, the removal efficiency is great; at the highest BGD concentration, it is poor. The percentage deletions that were the highest and lowest were 99.56% and 81.87%, respectively.

The maximum value was obtained at 50 mg/L and then decreases. When the initial dye concentration is low, there are more sites available than when it is high. As a result, removal efficiencies rise, and the majority of dye molecules are adsorbed by adsorbents at low initial dye concentrations. However, if the initial dye concentration is higher than that, the adsorbent’s active sites are fully retained, some dye molecules cannot be absorbed, and the removal efficiencies start to decline^31^.

### Design of the experiment and statistical evaluation of the findings

#### Analysis of variance

The statistical significance of each factor and their corresponding interactions in the obtained quadratic model was verified using analysis of variance (ANOVA), as is already indicated in Table 3. The current study’s empirical model for the removal of BGD has a high F-value of 327.22, indicating that the model is very reliable. Only 0.01% of the time, an F-value this high could be the result of noise. Additionally, the model’s P-value is extremely low (0.0001), demonstrating its relevance. Model terms are considered significant when the p-value is less than 0.0500^21^.

**Table 3:** Model summary statistics and suggested design for removal of BGD.

Source	Std. Dev	R^2^	Adjusted R^2^	Predicted R^2^	PRESS	
Linear	7.48	0.1795	0.0483	− 0.1941	2034.90	
2FI	8.39	0.2143	− 0.1993	− 0.2588	2145.09	
Quadratic	0.6089	0.9967	0.9937	0.9833	28.54	Suggested
Cubic	0.3859	0.9994	0.9975	0.9796	34.80	Aliased

The significance of the statistical model is indicated by its F-value of 327.22. The likelihood that an F-value this great may be caused by noise is 0.01%. A, B, C, D, AC, BC, BD, CD, A2, B2, and D2 are important model terms in this instance, but AB, AD, and C2 are not. The model is significant if the probability is greater than the F value of 0.0001. According to Mohamad et al.^32^, the likelihood of an F-value this significant occurring owing to noise is only 0.01%.

The model terms are not significant if the P-value is bigger than 0.0500. The developed empirical model’s lack of fit F-value is 2.94. This number suggests that, in comparison to the pure error, the lack of fit is not significant.

#### Model equation

The relationship between responses and independent parameters was explained using the final model equation that was obtained. The quadratic model was chosen because it successfully fits the statistics, as indicated in Table 4. Therefore, the second-order polynomial equation described the quadratic model that links the removal efficiency of BGD with independent process parameters, and Eq. (3.1) provides the percentage removal of BGD as a function of independent parameters (coded variables).

5 $$\begin{aligned} \% {\text{Removal }} = & \; + 98.66 - 1.30{\mathrm{*A}} + 0.8269{\mathrm{*B}} + 0.3469{\mathrm{*C}} - 3.20{\mathrm{*D}} \\ & \; - 0.0718{\mathrm{*AB}} + 0.5119{\mathrm{*AC}} - 0.0311{\mathrm{*AD}} + 1.06{\mathrm{*BC}} \\ & \; + 1.12{\mathrm{*BD}} + 1.03{\mathrm{*CD}} - 1.45{\mathrm{*A}}^{2} - 0.5762{\mathrm{*B}}^{2} \\ & \; + 0.0038{\mathrm{*C}}^{2} - 6.87{\mathrm{*D}}^{2} { } \\ \end{aligned}$$

**Table 4:** Variance analysis (ANOVA) for the quadratic model of BGD adsorption.

Source	Sum of squares	Df	Mean square	F-value	*p*-value	
Model	1698.52	14	121.32	327.22	< 0.0001	Significant
A-Adsorbent dosage	40.52	1	40.52	109.29	< 0.0001	
B-time	16.41	1	16.41	44.26	< 0.0001	
C-Initial BGD concentration	2.89	1	2.89	7.79	0.0137	
D-pH	246.12	1	246.12	663.82	< 0.0001	
AB	0.0825	1	0.0825	0.2225	0.6439	
AC	4.19	1	4.19	11.31	0.0043	
AD	0.0154	1	0.0154	0.0416	0.8411	
BC	17.86	1	17.86	48.17	< 0.0001	
BD	20.00	1	20.00	53.94	< 0.0001	
CD	17.01	1	17.01	45.88	< 0.0001	
A^2^	57.53	1	57.53	155.16	< 0.0001	
B^2^	9.11	1	9.11	24.56	0.0002	
C^2^	0.0004	1	0.0004	0.0011	0.9745	
D^2^	1292.88	1	1292.88	3487.02	< 0.0001	
Residual	5.56	15	0.3708			
Lack of Fit	4.75	10	0.4752	2.94	0.1230	Not significant
Pure error	0.8091	5	0.1618			
Cor total	1704.08	29				

A denotes the CCAC dosage, the contact time by B, the initial conc. BGD by C, and the pH by D. The impact of factors on the percentage elimination of BGD is seen in Eq. 2. While the positive coefficient values show that the factors have a positive impact on the adsorption of BGD, the negative coefficients generally reflect that the factors have a negative impact on the adsorption of BGD (raising the factors does not increase removal efficiency). In comparison to the effects of A, B, and C, D had a greater influence on the percentage elimination of BGD.

#### Model diagnostic plot

**Fig. 12:**
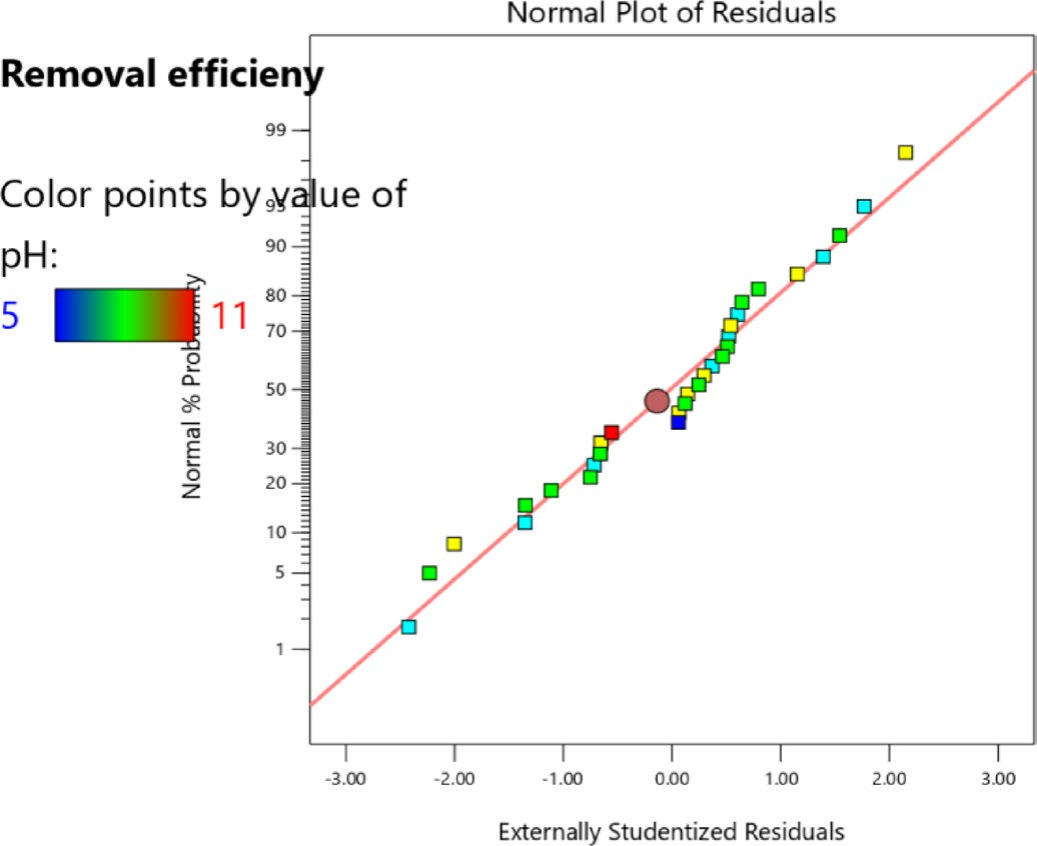
Normal % probability versus residuals plot for BGD removal.

An illustration of the model that can be used to decipher the random variation of the values is called a diagnostic plot. Figure 12 displays the residuals’ normal plot. The residuals were estimated along a straight line, indicating that the normality condition was met, since the normal probability plot showed that they follow a normal distribution. This shows that the quadratic polynomial model that was chosen for the BGD adsorption study is adequate.

**Fig. 13:**
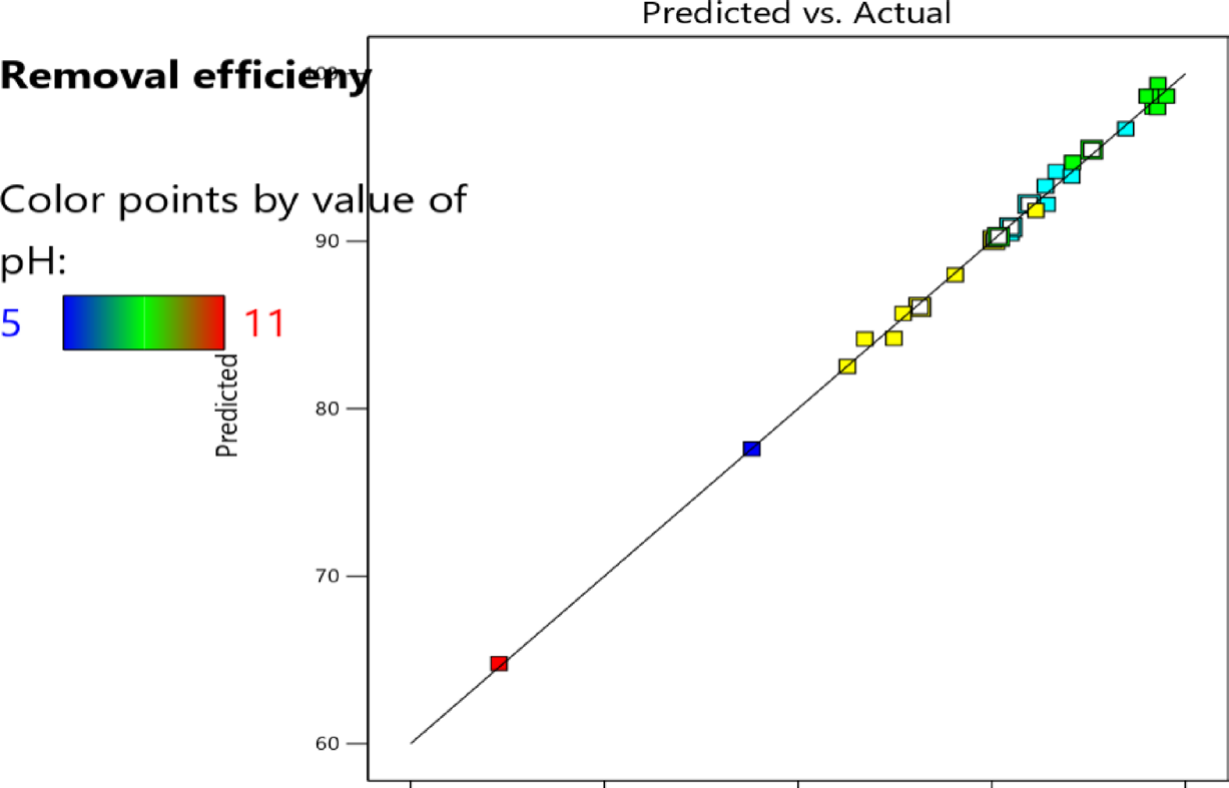
Predicted versus actual plot for BGD removal.

Figure 13 shows the extent to which the real and projected values agreed. The model’s predicted values were somewhat close to the straight line and very close to the experimental values. Consequently, the outcome shows that the actual values and the plotted anticipated values agreed well.

#### Interaction between parameters’ effects on BGD removal

If the independent factors interact to influence the dependent parameter, this is known as an interaction effect. The percentage removal of BGD in this investigation was found to be significantly impacted by the combined effects of adsorbent dose and initial BGD concentration, contact time and first BGD concentration, contact time and pH, initial concentration, and pH.

- The effects of the initial BGD concentration and CCAC dosage (AC) interaction

For larger concentrations of BGD solutions, the greatest adsorption was seen at lower adsorbent dosages. However, as the adsorbent dose increases, the adsorption decreases due to the increased dose concentrations. Unsaturation of the adsorption sites could be the cause of this decline. Larger BGD concentrations resulted in a larger percentage of dye removal effectiveness, according to CCAC adsorption experiments.

At 69.65 mg/L of BGD solution and 0.2019 g of adsorbent, the maximum removal efficiency was 99.76%. Because there was more contact between the adsorbate and the adsorbent’s accessible sites, the removal efficiency was higher. The dye’s resistance to mass transfer between the aqueous and solid phases may be resolved by increasing the initial adsorbent concentration^33^.

b. The interaction of contact time and initial Concentration of BGD (BC)

**Fig. 14:**
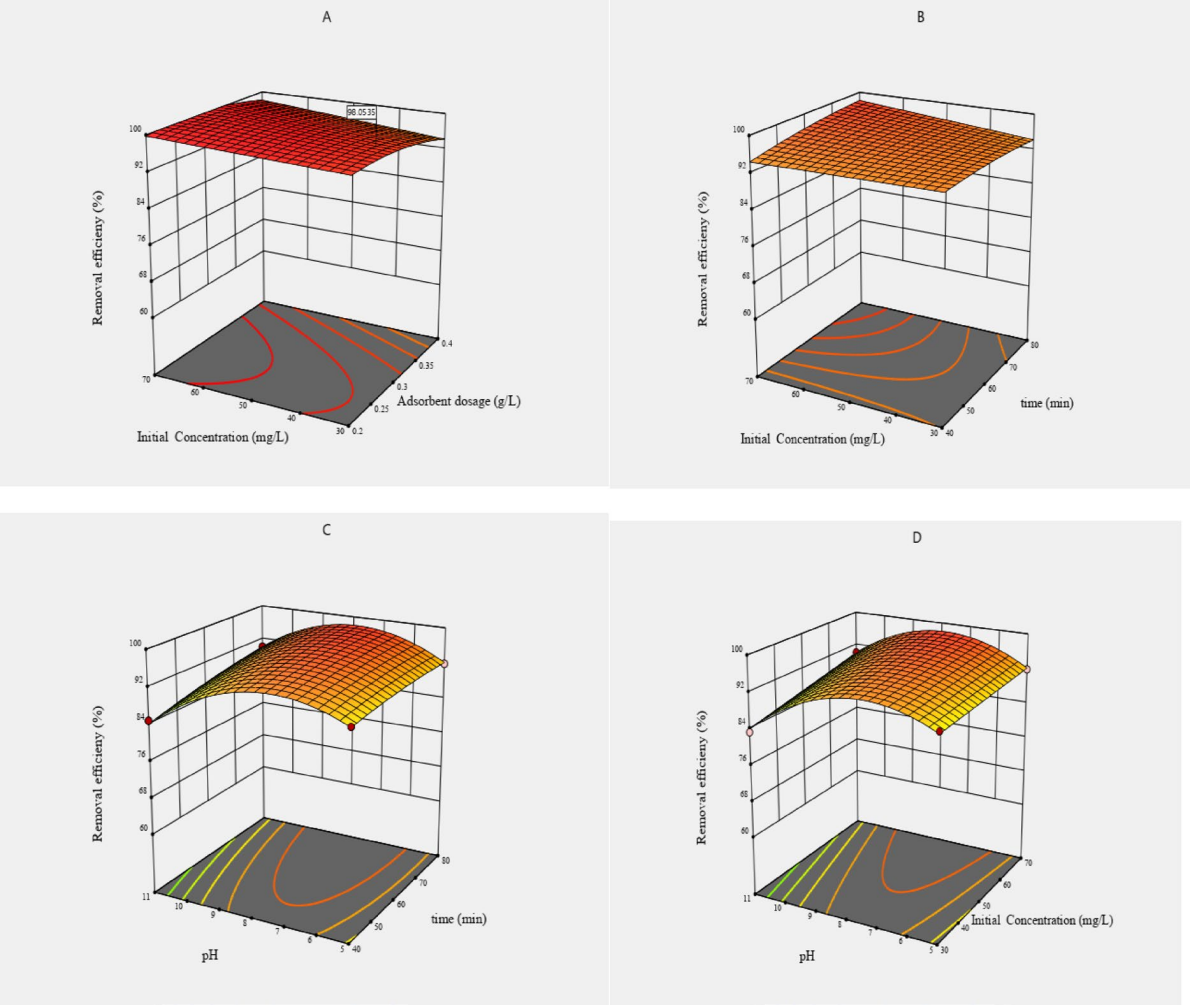
3D plot for the combined effects of (**A**) initial concentration of BGD and Adsorbent dosage (**B**) Initial Concentration and contact time (**C**) contact time and pH (**D**) pH and initial concentration on BGD removal.

Figure 14B illustrates how the initial BGD concentration and contact time interact to affect BGD elimination. According to the diagram, the percentage of BGD removed rises with an increase in both the initial BGD concentration and contact duration. At high initial concentrations, the removal efficiency of BGD increased as contact time increased, and when contact time exceeded the optimal value, it decreased.

The absence of a constant number of adsorption active sites on the adsorbent’s surface caused the removal efficiency to decrease as the initial concentration increased. This is because, at high dye concentrations, the ratio of surface-active ion sites to total molecules is low, causing all molecules in the solution to adhere to the CCAC dosage surface. At an initial concentration of 69.52 mg/L BGD and a contact period of 40.71 min, a maximum removal percentage of 94.55% was achieved.

Hence, the adsorption rate was higher at the initial contact time, as enough active sites were available for adsorption. However, through time, a higher amount of BGD was adsorbed on the CCAC dosage surface, and therefore the surface area decreased Vahab Ghalehkhondabi^34^ observed similar studies.

c. Contact time and pH (BD) interaction effects

Figure 14C illustrates how pH and contact time interact to remove BGD. The rationale based on this model states that as contact time increases and solution pH decreases, the effectiveness of BGD removal rises. At pH, 6.98 and contact time 65.69 min, the CCAC’s maximum removal effectiveness (99.09%) was observed, while the other two variables were at their medium level. Because BG is a basic dye, the removal efficiency steadily drops at increasing pH values.

To reach equilibrium time, when the saturation of the active sites prevents additional adsorption, the percentage may remain constant during the adsorption process. According to Mohamad et al.^32^, saturation occurs when the dye particles completely bind to all of the adsorbent’s active sites.

d. The effects of pH and the initial BGD concentration interact (CD)

The interaction effect of the initial concentration of BGD and pH on BGD removal is shown in Fig. 14D. The maximum removal efficiency of the CCAC was found at pH 7.06, and at higher pH values, the removal efficiency decreases gradually because BGD is a basic dye. Adsorption studies of CCAC show that the percentage of dye removal efficiency was higher at a higher concentration of BGD. The removal efficiency is probably due to increased contact of adsorbate with available sites of adsorbent. This can be due to the increase of the initial adsorbent concentration being a driving force to resolve the resistance of the dye to mass transfer between the aqueous and solid phases. The removal efficiency was 96.5% at a pH of 7.06 and a concentration of 55.44 mg/L.

#### The optimization process of adsorption for BGD removal

CCD was used to optimize the effects of process variables, including adsorbent dosage, beginning BGD concentration, pH, and contact duration, on the percentage removal of BGD. Table 5 listed goals for each variable’s numerical optimization. Following optimization, studies were conducted to validate the results of the experiment.

**Table 5:** Process parameters and goals for optimization.

Name	Goal	Lower limit	Upper limit	Importance
A:Adsorbent dosage	Is in range	0.2	0.4	3
B:time	Is in range	40	80	3
C:Initial BGD concentration	Is in range	30	70	3
D:Ph	Is in range	5	11	3
Removal efficiency	Maximize	64.565	99.02	3

The numerical optimization provided optimum points for independent variables that might maximize the response while considering the desirability function. The examined independent process parameters, including the initial BGD concentration of 59.463 mg/L, the adsorbent dose of 0.276, the pH of 7.172, and the contact duration of 63.847 min, were shown to have optimal points among the derived solutions using numerical optimization.

It was discovered that the greatest removal effectiveness of BGD ions was 99.390 percent at these independent parameter optimal points.

**Fig. 15:**
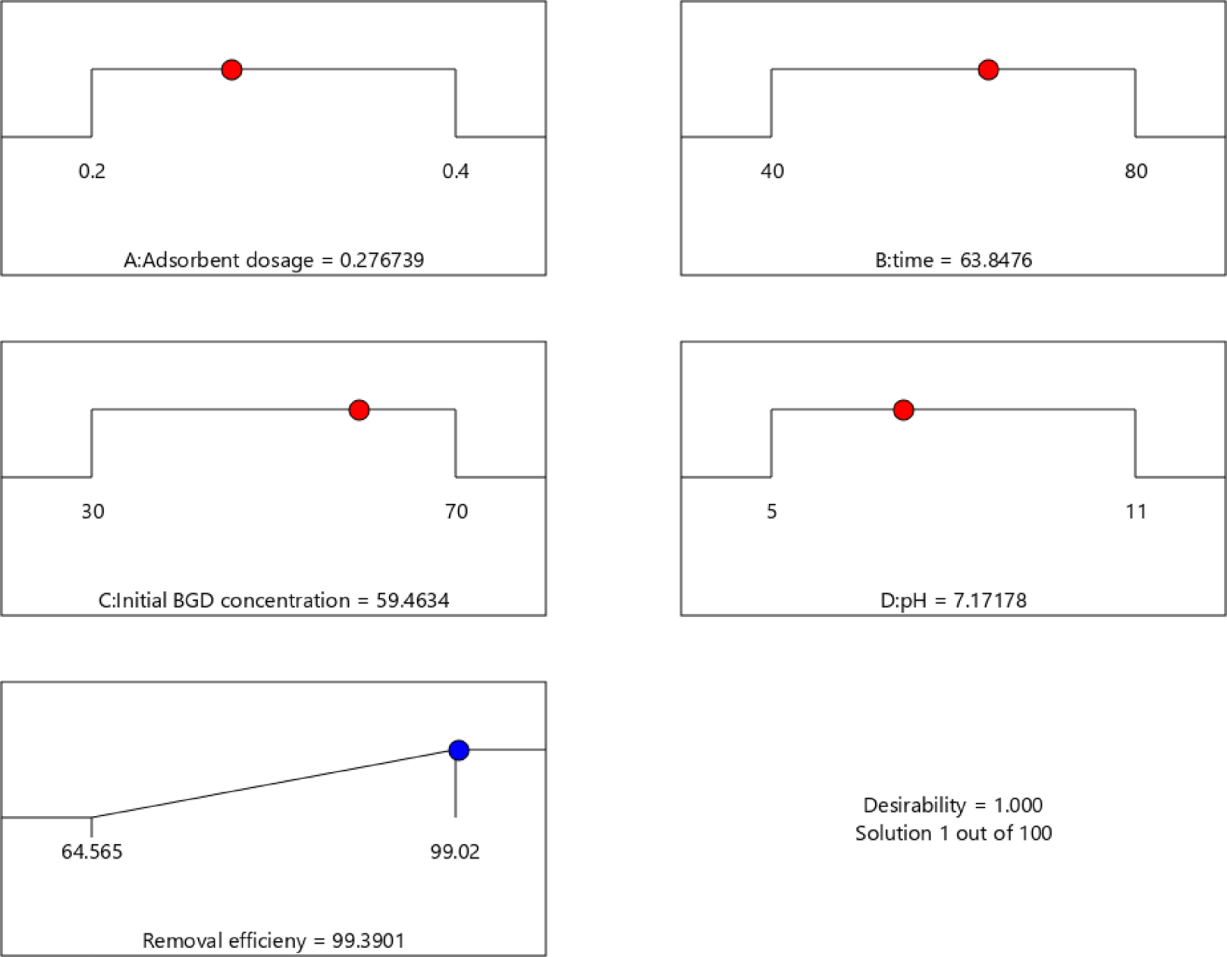
Optimal operational parameter values for BGD removal.

In Fig. 15, the ideal conditions chosen were depicted on ramps. On the ramps connecting the process variable values, the optimal spots responsible for the best removal efficiency were marked. When the adsorbent dosage was 0.276 g/100 ml, the initial BGD concentration was 59.463 mg/1000 ml, the solution pH was 7.172, and the contact period was 63.847 min, the ideal removal efficiency indicated by the on-ramps was thus achieved.

### Adsorption isotherms

**Fig. 16:**
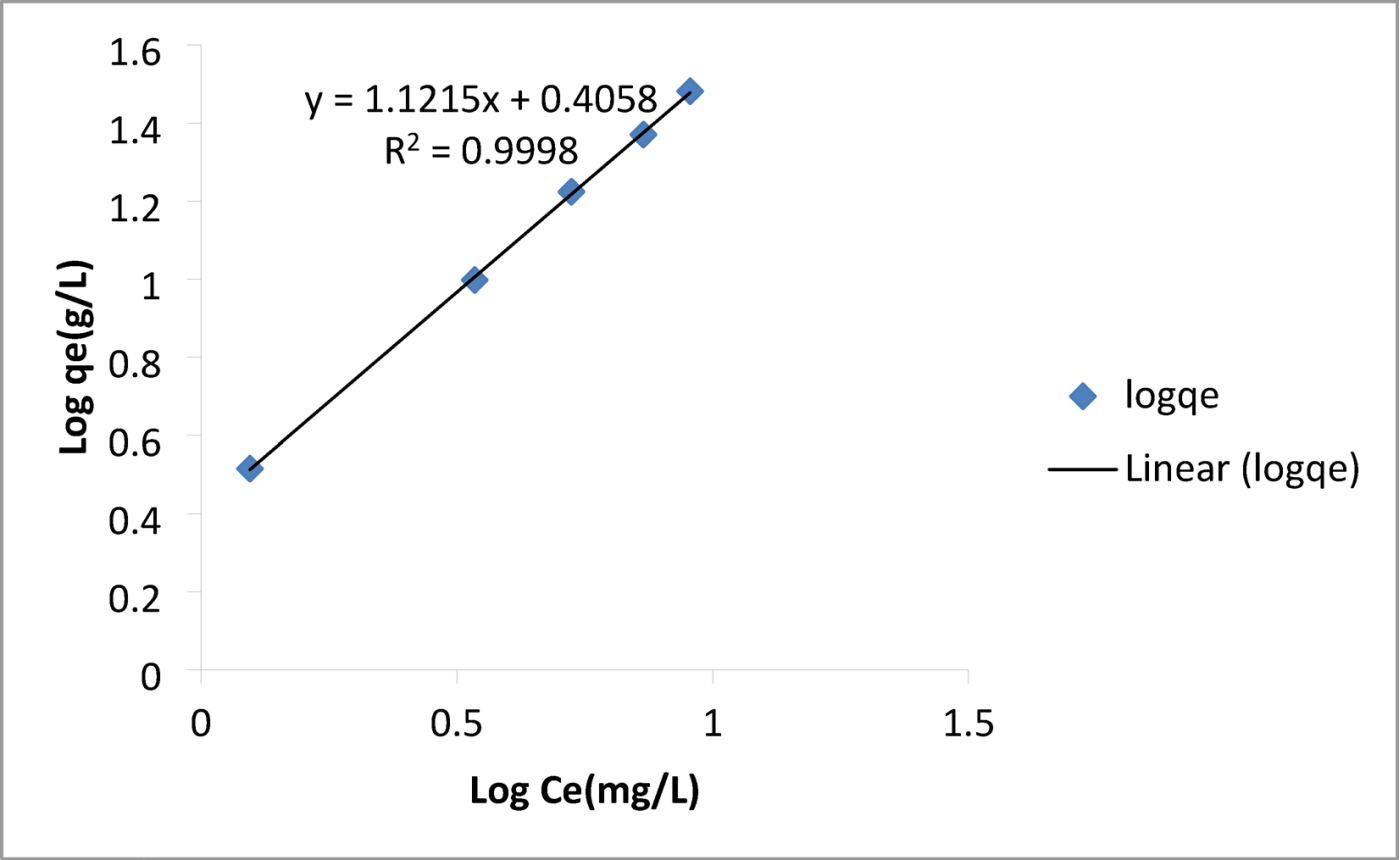
Languimuir isotherm model of BGD removal.

The studies of adsorption isotherm were performed by differing initial concentrations of BGD from 10 to 90 mg/L at room temperature. Using 100 mL of initial BGD solutions with an adsorbent dose of 0.267 g, a shaking speed of 200 rpm, a contact time of 63.847 min, and a pH of 7.172. A Langmuir model and Freundlich isotherm models, as shown in Fig. 16, interpreted the adsorption behaviour between the CCAC and BGD ions under different concentrations. The outcomes set in Table 6 show the experimental data were fitted with both the Langmuir and Freundlich adsorption isotherm models. However, Fig. 16 describes that the result from experimental data fitted very well with the Freundlich isotherm model but not the Langmuir isotherm. Thus, it indicates that the adsorption of BGD ions on the surface of CCAC effectively undergoes the monolayer and homogeneous adsorption process of BGD ions rather than the multilayer adsorption process.

**Table 6:** Optimum points.

Number	Adsorbent dosage	Contact time	Initial BGD concentration	pH	Removal efficiency	Desirability	
1	0.276	63.847	59.463	7.172	99.39	1.000	Selected

The values of separation factor RL (0 < RL < 1) given in Table 7 designate the favourability of the adsorption process with a maximum adsorption capacity (qmax) of 98.32 mg*g⁻^1^ (Table 7). Furthermore, a value of the Langmuir coefficient (b), which is 0.026 L/mg, implies strong binding sites exist on the surface of the adsorbent; thus, the adsorbents can adsorb dyes effectively.

**Table 7:** Langumir and Freundlich isotherm models.

Adsorbent	Languimer			Freundlich			
CCAC	Qmax (mg/g)	b (l/mg)	R^2^	K_f_	N	1/n	R^2^
	98.2	0.026	0.9226	2.54	0.89	1.1215	0.9998

On the other hand, as represented in Table 7, the value of the Freundlich constant, (n) which is greater than 1, indicates favourable adsorption.

A coefficient (R2) of 0.999 representing that heterogeneous conditions can exist.Generally, from this we conclude that the Freundlich isotherm model gives the best fit to the experimental data in contrast to the Langmuir isotherm model with a maximum correlation coefficient as indicated in Table 7 below. This is the same with the study of Fodeke & Olayera^35^ (Tables 8 and 9).

**Table 8:** Langmuir separation factor (R_L_).

Co	10	30	50	70	90
R_L_	0.79	0.56	0.43	0.35	0.04

**Table 9:** Comparing several adsorbents for the elimination of BGD.

Adsorbent	Co (mg/L)	Adsorption capacity (mg/g)	Removal efficiency	References
Pyrus pashia leaves	10		93.35	Fiaz et al.^28^
Activated carbon from guava seeds	10	80.5	98	Mansour et al.^26^
Fava bean peels (FBP)	5	28.14	84	Nahali et al.^27^
Lawsonia inermis seed powder	20	34.96	93%	Ahmad and Ansari^25^
Corn cobs activated carbon	59.46	98.04	99.309	This study

## Conclusions

In order to adsorb BGD from aqueous solution, CCAC adsorbents were made in this study utilizing H_2_SO_4_ activation. The removal of BGD was examined utilizing a variety of factors; the removal of BGD was greatly impacted by pH, contact time, CCAC dosage, and beginning concentration. The adsorption of BGD from an aqueous solution was optimized using the RSM with CCD.

According to the results of the analysis, the quadratic model’s optimal adsorption parameters for the highest removal effectiveness of BGD were 7.172, 59.463 mg/L, 0.276 g, and 63.847 min for pH, initial BGD concentration, CCAC dosage, and contact duration, respectively. The maximal removal efficiency of BGD was determined to be 99.39% at these ideal process settings. Overall, this investigation demonstrated that CCAC was a biomass adsorbent that effectively removed BGD.

## Data Availability

The raw data sets used and analyzed are available from the correspondence author at a reasonable request at any time.
